# The Dispensaries of Assam

**Published:** 1904-08-27

**Authors:** 


### THE DISPENSARIES OF ASSAM.

The latest report of the dispensaries of Assam is particularly satisfactory, because it discloses two important facts, a greater yearly increase in the number of patients treated and a lower death-rate. On the first count the gradual ascent of the figures for the last four years cannot be otherwise than gratifying to those concerned in the hospital administration. In 1899, 623,803 patients; in 1900, 682,989; in 1901, 734,682; and in 1902, 821,331 came under medical care, and the rise was not only in admissions to the hospitals, but also in those who attended the out-patients' departments. With regard to the latter, notwithstanding that so much is done to emphasise the necessity of personal attendance, as many as 276,707 persons were represented by their friends during 1902. But the move, slight though it is, is in the right direction, for the figures in 1901 show that of 100 so-called attendances only 65 were personal, whilst in 1902 the number had risen to 66. As to the decrease in the number of deaths, out of 6,683 patients treated only 11 per cent. died in hospital, the percentage for the previous year being over 12 per cent. These figures include the deaths amongst paupers and destitute coolies, many of whom were sent in—notably from the municipality of Sylhet—in an almost moribund state. Their serious condition is apparent from the fact that out of 51 who were brought into the hospital in 1902, 25 died. If these cases were omitted the death-rate amongst in-patients would be only 8·34, the corresponding ratio for the year before being 9·87. This is undoubtedly a sign of better medical skill and better nursing. The total number of dispensaries in the province of Assam was 133 in 1902. In the new hospital at Lungleh 233 patients were treated, whilst two small dispensaries, which were established for the treatment of kala-azur cases were closed, owing to the decline of that disease. One dispensary at Gauripur in the Goalpara district was taken over by the Raja of that place at the end of the year. Small-pox was very prevalent during 1902, there being 2,571 cases. At the Hailakandi sub-divisional dispensary no fewer than 300 persons were treated for small-pox. In this subdivision inoculation is freely practised by Ganaks, and there is no doubt that this practice of inoculation is the cause of the greater prevalence of small-pox here than elsewhere, and it is much to be desired that, whether or not it is found feasible to make vaccination compulsory, measures should be taken as soon as possible to render the operation of inoculation illegal. The number of surgical operations, both principal and secondary, rose from 15,000 to 17,000, and the number of deaths after operation was only 9, a most satisfactory figure, more especially as the record for the two previous years showed 27 and 41 respectively. Statistics prove that the assistant surgeons are now taking up the extension of surgery in earnest, and, as the civil surgeons are too much engaged in out-station inspection of dispensaries and tea-gardens to do nearly as much work in the direction of surgery as their confrères in Bengal and the United Provinces, it is on the assistant surgeons in particular that the patients rely for operative skill. The number of women attending the dispensaries is disappointing. It was hoped that by securing greater privacy the female population of the province would be anxious to come when they were out of health. In some cases the reluctance has been traced to the unpopularity of the hospital assistants in charge, but generally speaking it seems due to the timidity and feeling of the women themselves. The provision of separate consulting rooms with separate entrances for females has not produced any appreciable increase in numbers. In some of the hill districts the women object to enter them at all, and will not even sleep in the wards set apart for them. They do not mind in the very least the proximity of men of their own race, but express themselves as "quite sure they won't sleep apart in rooms by themselves." As to diet, by far the largest proportion of in-patients were dieted from the dispensary funds, at an average cost of Rs. 0.2.9 or a fraction over twopence, but 1,097 dieted themselves. Respecting income and expenditure the total income of the dispensaries was Rs. 2,55,098. The grants from local funds came to Rs. 94,000, from Government to Rs. 83,000, from natives to nearly Rs. 15,000, and from Europeans to Rs. 5,000. The total expenditure was Rs. 2,03,678, leaving a balance of Rs. 51,420. The increase of expenditure was mainly on account of salaries of medical officers, etc., consequent upon the opening of three more dispensaries.

